# Store-Operated Calcium Channels Control Proliferation and Self-Renewal of Cancer Stem Cells from Glioblastoma

**DOI:** 10.3390/cancers13143428

**Published:** 2021-07-08

**Authors:** Elodie Terrié, Nadine Déliot, Yassine Benzidane, Thomas Harnois, Laëtitia Cousin, Patrick Bois, Lisa Oliver, Patricia Arnault, François Vallette, Bruno Constantin, Valérie Coronas

**Affiliations:** 1 CNRS ERL 7003, Signalisation et Transports Ioniques Membranaires, University of Poitiers, CEDEX 09, 86073 Poitiers, France; elodie.terrie@univ-poitiers.fr (E.T.); nadine.deliot@univ-poitiers.fr (N.D.); benzidane.yassine@yahoo.fr (Y.B.); thomas.harnois@univ-poitiers.fr (T.H.); laetitia.cousin@univ-poitiers.fr (L.C.); patricia.arnault@univ-poitiers.fr (P.A.); bruno.constantin@univ-poitiers.fr (B.C.); 2 EA 4379, Signalisation et Transports Ioniques Membranaires, University of Poitiers, CEDEX 09, 86073 Poitiers, France; patrick.bois@univ-poitiers.fr; 3 CRCINA-UMR 1232 INSERM, Université de Nantes, CEDEX 01, 44007 Nantes, France; Lisa.Oliver@univ-nantes.fr (L.O.); Francois.Vallette@univ-nantes.fr (F.V.); 4 CNRS GDR3697, Micronit “Microenvironment of Tumor Niches”, 37000 Tours, France

**Keywords:** glioblastoma, glioma, cancer stem cell, calcium channel, store-operated channel, TRPC, Orai, STIM

## Abstract

**Simple Summary:**

Glioblastoma is a high-grade primary brain tumor that contains a subpopulation of cells called glioblastoma stem cells, which are responsible for tumor initiation, growth and recurrence after treatment. Recent transcriptomic studies have highlighted that calcium pathways predominate in glioblastoma stem cells. Calcium channels have the ability to transduce signals from the microenvironment and are therefore ideally placed to control cellular behavior. Using multiple approaches, we demonstrate in five different primary cultures, previously derived from surgical specimens, that glioblastoma stem cells express store-operated channels (SOC) that support calcium entry into these cells. Pharmacological inhibition of SOC dramatically reduces cell proliferation and stem cell self-renewal in these cultures. By identifying SOC as a critical mechanism involved in the maintenance of the stem cell population in glioblastoma, our study will contribute to the framework for the identification of new therapies against this deadly tumor.

**Abstract:**

Glioblastoma is the most frequent and deadly form of primary brain tumors. Despite multimodal treatment, more than 90% of patients experience tumor recurrence. Glioblastoma contains a small population of cells, called glioblastoma stem cells (GSC) that are highly resistant to treatment and endowed with the ability to regenerate the tumor, which accounts for tumor recurrence. Transcriptomic studies disclosed an enrichment of calcium (Ca^2+^) signaling transcripts in GSC. In non-excitable cells, store-operated channels (SOC) represent a major route of Ca^2+^ influx. As SOC regulate the self-renewal of adult neural stem cells that are possible cells of origin of GSC, we analyzed the roles of SOC in cultures of GSC previously derived from five different glioblastoma surgical specimens. Immunoblotting and immunocytochemistry experiments showed that GSC express Orai1 and TRPC1, two core SOC proteins, along with their activator STIM1. Ca^2+^ imaging demonstrated that SOC support Ca^2+^ entries in GSC. Pharmacological inhibition of SOC-dependent Ca^2+^ entries decreased proliferation, impaired self-renewal, and reduced expression of the stem cell marker SOX2 in GSC. Our data showing the ability of SOC inhibitors to impede GSC self-renewal paves the way for a strategy to target the cells considered responsible for conveying resistance to treatment and tumor relapse.

## 1. Introduction

Glioblastoma (GBM) is a high-grade primary brain tumor and ranks among the most lethal of all human cancers. Treatment for patients includes maximal tumor resection followed by radiotherapy with concomitant chemotherapy with temozolomide. Despite this multimodal treatment, glioblastoma remains a refractory malignancy with 90% of patients experiencing tumor relapse and with a median survival of barely 15 months from the time of diagnosis [1]. In addition to being highly infiltrative, GBM exhibit complex cellular and molecular heterogeneity, both between patients and within individual tumors, which represents a major hurdle for an effective treatment and leads to nearly systematic recurrence. In an effort to facilitate the development of more efficient therapies, large-scale molecular studies have classified GBM into three major subtypes according to their molecular profiles associated with prognostic values: mesenchymal, pro-neural/neural, and proliferative/classical subtypes [2,3]. The pro-neural subtype is the most favorable subtype for patients compared to the mesenchymal and classical subtypes [4]. At the cellular level, the poor response of GBM to treatment and tumor relapse have been ascribed to the persistence of a rare population of cells that, like physiological stem cells, are endowed with regenerative potential and have therefore been termed glioblastoma stem cells (GSC) [5]. Resistant to the current treatments, GSC possess self-renewal capacities and can rebuild the broad spectrum of cells that form the tumor. Accordingly, a complete cure of GBM would require the eradication of these tumor initiating and propagating cells [5].

A comparative analysis of transcriptomic signatures in stem- and non-stem-GBM cells identified an enrichment of Ca^2+^ signaling genes in cells displaying stem cell characteristics, suggesting a potential role for Ca^2+^ in GSC [6]. The Ca^2+^ ion is a ubiquitous second messenger whose variations of its cytosolic concentration regulate a wide range of cellular processes, including cell proliferation, migration and death [7]. Many extracellular signals from the microenvironment induce Ca^2+^ responses by promoting Ca^2+^ release from the internal stores and Ca^2+^ influx from the extracellular environment. In non-excitable cells, store-operated channels (SOC) are one of the predominant pathways for Ca^2+^ entry [8]. Depending on the cell types, SOC are formed by Orai, mainly Orai1 that supports the I_CRAC_ (the Ca^2+^-release-activated Ca^2+^ current), and TRPC proteins, mainly TRPC1 (Transient Receptor Potential Canonical 1) that cooperates with Orai1. STIM1 (Stromal Interaction Molecule 1), the endoplasmic reticulum (ER) Ca^2+^ sensor, directly activates Orai1 [8] and may also interact with TRPC1 [9].

Store-operated Ca^2+^ entries (SOCE) are usually obtained after stimulation of various plasma membrane receptors that activate phospholipase C. This enzyme then hydrolyzes phosphatidylinositol-4,5-bisphosphate (PIP2) to diacylglycerol (DAG) and inositol triphosphate (IP3). DAG can activate protein kinase C (PKC) that opens receptor-operated channels (ROC) of TRPC family (e.g., TRPC3/6). The other second messenger IP3 binds to the IP3 receptor (IP3R) located on the ER membrane and induces Ca^2+^ release from the ER stores into the cytosol (see the graphical abstract). Upon sensing a drop in ER Ca^2+^ levels, STIM1 oligomerizes, undergoes conformational changes, and then opens SOC, triggering an influx of Ca^2+^ from the extracellular space into the cytosol of the cell. This Ca^2+^ influx, which may be long-lasting, is essential both for the replenishment of the ER Ca^2+^ pool and for the recruitment of Ca^2+^-related signal transduction actors [8].

Transcriptomic analysis of brain tumor tissue from patients suffering from GBM revealed an overexpression of SOC (TRPC1, Orai1) and their ER activator, STIM1 [10,11]. Furthermore, studies on conventional GBM cell lines, mainly maintained in serum-based media, have shown that SOC promote proliferation and invasion [12,13,14], although there is some possible controversy about this [15]. Yet, the possible role of SOC in GSC, the subset of cells considered as responsible for tumor initiation, growth and relapse, is still unclear [16]. As our previous work demonstrated that SOC regulate the self-renewal of adult neural stem cells in the subventricular zone [17], the cells that represent the possible cells of origin of GSC [18], we set-up the present study to examine the presence and role of SOC in GSC. Using multiple approaches, our data demonstrate that indeed, GSC express SOC that support Ca^2+^ entries whose pharmacological inhibition reduces GSC proliferation and self-renewal, thereby diminishing the stem cell population.

## 2. Materials and Methods

### 2.1. Solutions and Chemicals

SKF-96365 and YM-58483 (also called BTP2) were purchased from Sigma Aldrich (Saint-Louis, MO, USA) and were dissolved in water and DMSO, respectively. GSK-7975A was purchased from AOBIOUS and was dissolved in DMSO. Cell culture media and growth factors were purchased from Invitrogen (Carlsbad, CA, USA).

### 2.2. GSC Cell Culture

Primary cultures were previously derived from five different GBM surgical specimens [19] and were grown in a defined medium used for GSC culture (DMEM/HAM-F12, 2 mmol·L^−1^ glutamine, N2 and B27 supplement, 2 μg·mL^−1^ heparin, 20 ng·mL^−1^ EGF and 25 ng·mL^−1^ bFGF, 100 U·mL^−1^ penicillin and 100 μg·mL^−1^ streptomycin). These conditions allowed the development of floating spheres that were collected, dissociated, and reseeded weekly. All experiments were performed with cells that were subjected to less than 10 passages.

### 2.3. Immunoblotting

For Western blotting analysis, 10^6^ cells were lysed in Laemmli loading buffer (Sigma Aldrich). The cell extract was then sonicated and heated for 5 min at 95° C. Cell lysates were separated by SDS-PAGE using 9% Bis-Tris polyacrylamide gels. Proteins were transferred to Amersham Protran nitrocellulose membranes (0.20 μm-pore size; GE Healthcare, Little Chalfont, UK). Immunoblots were probed overnight at 4 °C in TBS-Tween (100 mmol·L^−1^ Tris–HCl, 150 mmol·L^−1^ NaCl and 0.1% Tween-20, pH 7.6) with 3% fat milk with one of the antibodies listed in Table S1. Membranes were washed and incubated for 1 h at 4 °C with anti-rabbit or anti-mouse horseradish peroxidase-conjugated secondary antibodies (1:5000, GE Healthcare, Little Chalfont, UK). Membranes were washed three times for 5 min per wash with TBS-Tween, and bound antibodies were detected using ECL chemiluminescent substrate (Immobilon, Millipore, Billerica, MA, USA). Results were analyzed with GeneGnome XRQ (SYNGENE Ozyme, Cambridge, UK). Quantification was performed using GeneTools from syngene. SOX2 expression was normalized to GAPDH for each sample, and then the normalized SOX2 expression in the treated samples was expressed relative to DMSO (control) treatment.

### 2.4. Immunostaining

For immunostaining, GBM spheres were projected on coverslips using a cytospin device and fixed in methanol at −20 °C for 10 min. After permeabilization and blocking of non-specific binding sites, preparations were stained with one of the antibodies listed in Table S1 and then incubated with the appropriate Alexa fluor 555 or Alexa fluor 488 conjugated antibodies before analysis with a spectral confocal FV-1000 station installed on an inverted microscope IX-81 (Olympus). DAPI was used to label the nuclei. Emitted fluorescence was detected through spectral detection channels between 425–475 nm, 500–530 nm and 550–625 nm, for UV, green and red fluorescence, respectively, and through a 650 nm long pass filter for far-red fluorescence.

### 2.5. Intracellular Ca^2+^ Measurements

Cells were plated at 75,000 to 150,000 cells·mL^−1^ on fibronectin coated glass coverslips. After 4 h, cells were incubated for 30 min at 37 °C with 6 μmol·L^−1^ of the ratiometric Ca^2+^ sensitive probe Fura-2-AM (Santa Cruz Biotechnology). Assays were performed in standard external buffer (130 mmol·L^−1^ NaCl, 5.4 mmol·L^−1^ KCl, 0.8 mmol·L^−1^ MgCl_2_, 10 mmol·L^−1^ HEPES, 5.6 mmol·L^−1^ D-glucose, pH 7.4) completed with 1.8 mmol·L^−1^ Ca^2+^ or 0.1 mmol·L^−1^ EGTA for the Ca^2+^-free solution. SOCE was triggered with thapsigargin (Sigma Aldrich, 4 μmol·L^−1^) and SOCE/ROCE, with sphingosine-1-phosphate (Sigma Aldrich, 1 μmol·L^−1^). Fluorescence images were recorded using a lambda 421 beam combiner coupled to an inverted microscope Olympus IX73 and Zyla sCMOS camera 4.2 PLUS. Consecutive excitation at 340 nm and 380 nm was undertaken every 2 s, and emission fluorescence was collected at 505 nm at 37 °C, using the Metafluor software. The fluorescence ratio 340/380 nm was measured over time in selected regions of interest (ROI) on several cells and normalized to the basal fluorescence obtained before stimulation.

### 2.6. Cell Proliferation Assays

Cell proliferation was evaluated by bromodeoxyuridine (BrdU) incorporation in DNA using an ELISA assay. GBM spheres were dissociated as single cells that were plated at the concentration of 30,000 cells·mL^−1^ in polylysine-coated 96-well plates and treated for 24 h with SKF-96365, YM-58483, GSK-7975A or DMSO. BrdU was added to the medium for the last 4 h of the assay before quantification of BrdU incorporated into DNA according to the manufacturer’s instructions (Roche diagnostics, Meylan, France) [20,21].

### 2.7. Sphere Forming and Self-Renewal Assays

GBM spheres were dissociated and seeded at 1000 cells per well in 24-well plates in the cell culture medium alone (control for SKF-96365) or supplemented with either SKF-96365 (3 μmol·L^−1^), YM-58483 (10 μmol·L^−1^), GSK-7975A (20 μmol·L^−1^) or DMSO (the dilution buffer of YM-58483 and GSK-7975A). After a 7-day incubation period, primary spheres were counted under the microscope. For self-renewal assays, the cells were collected, centrifuged and reseeded at 1000 cells per well in culture medium to obtain secondary spheres.

### 2.8. Limit Dilution Assays

Limit dilution assays were performed as described previously [22]. GBM spheres were dissociated and seeded in 200 µL of culture medium at decreasing concentrations (from 400 to 1 cell per well) into 96-well plate. After 7 days, the number of wells without spheres was counted and related to the number of cells plated per well. Based on a Poisson distribution of cells, the frequency of GSC corresponds to the number of cells for which 37% of the wells are empty [22].

### 2.9. SOX2 Expression Assay

GBM spheres were dissociated as single cells and then treated for 7 days with YM-58483 (10 μmol·L^−1^) or GSK-7975A (20 μmol·L^−1^) or their solvent (DMSO) used as control. After this period, immunoblotting of SOX2 was performed as described above.

### 2.10. Statistics

Statistics were performed using GraphPad prism. Mann–Whitney tests were used for 2-by-2 comparison and ANOVA for the proliferation assay. Statistical significance level was set for *p* values < 0.05 and represented on the figures by: * for *p* < 0.05, ** for *p* < 0.01, *** for *p* < 0.001, **** for *p* < 0.001.

## 3. Results

### 3.1. Patient-Derived GSC Express Functional SOC

**Figure 1:**
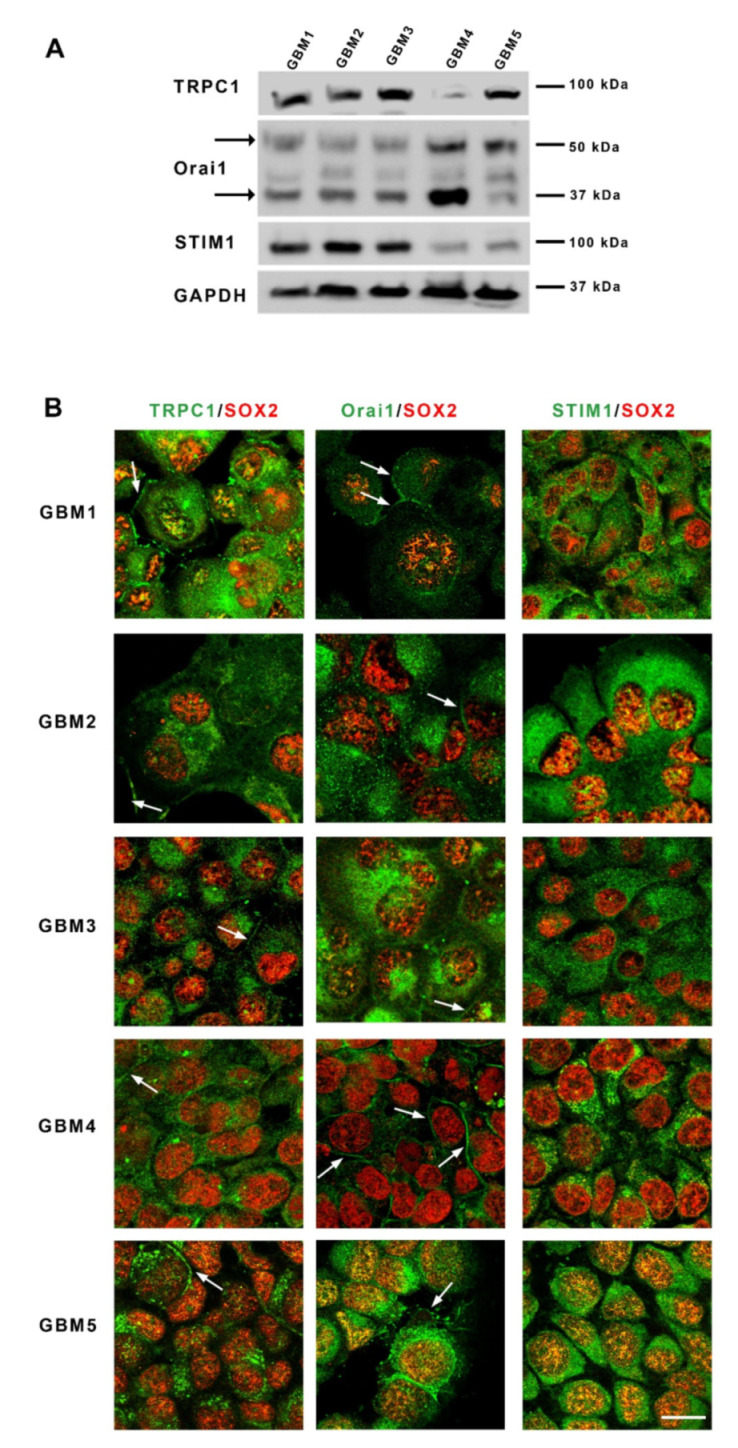
Glioblastoma stem cells express store-operated channels Orai1 and TRPC1 and their activator STIM1. (**A**): Immunoblotting of TRPC1, Orai1 and STIM1 proteins from five different primary cultures of GSC (GBM1-GBM5) derived from patients with GBM. Arrows point to the two bands obtained for Orai1 that correspond to the unglycosylated (37 kDa) or the glycosylated (50 kDa) form of Orai1. (**B**): Micrographs depicting co-labeling of TRPC1, Orai1 or STIM1 (green) with SOX2, a stem cell marker (red), in the five GBM cultures. Arrows indicate the membrane localization of the labeling. Scale bar: 25 µm. Abbreviations: STIM1, stromal interaction molecule 1; TRPC1, transient receptor potential canonical 1; kDa, kiloDalton.

The study was performed on five different primary cultures previously derived from GBM surgical specimens [19] and grown in a defined medium used for GSC culture. All cultures expressed SOX2, a protein known to be involved in stemness maintenance in GSC (Figure 1B) [23]. GBM1, GBM2 and GBM3, which display a mesenchymal phenotype, were additionally positive for CD44, whereas GBM4 and GBM5 expressed CD133, as expected for non-mesenchymal GBM cells (Supplementary Figure S1).

To investigate whether GSC expressed the main protein components supporting store-operated Ca^2+^ entries, we examined in these cells grown as spheres, the presence of both TRPC1 and Orai1 channels as well as their ER activator STIM1. Western blot analysis showed that all cultures expressed detectable levels of Orai1, TRPC1 and STIM1, although at different levels (Figure 1A). Of note, glycosylated Orai1 (MW 50 kDa), known for its localization to the plasma membrane and for its role in supporting calcium influx, was detected in all cells, as well as the unglycosylated form [24,25] of Orai1 (MW 37 kDa). Immunostaining of GSC cultures confirmed the expression of these SOC components in GSC and showed that Orai1 and TRPC1 labeling can be intracellular and also found as a strong labelling at the periphery, suggesting localization at the plasma membrane in SOX2-expressing GSC (Figure 1B). STIM1 staining was only intracellular and often displayed a punctuated pattern characteristic of localization in the endoplasmic reticulum (ER) (Figure 1B).

**Figure 2:**
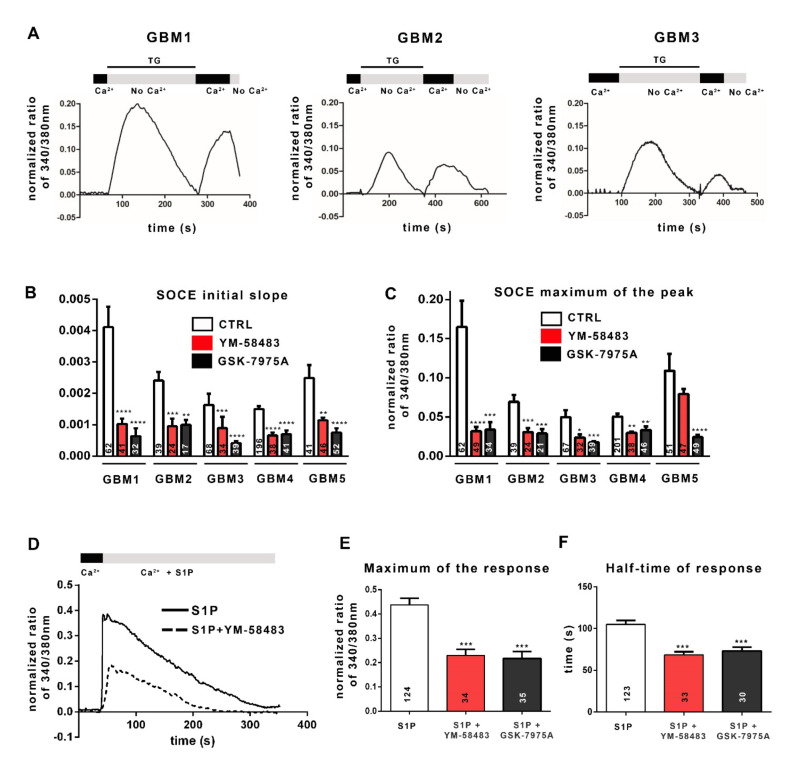
Cancer stem cells from GBM display SOCE. (**A**): Examples of representative traces of SOCE recorded in GSC cultures. The traces shown here have been recorded in GBM1 cells, GBM2 cells and GBM3 cells. To obtain these traces, cells were loaded with Fura-2AM and then incubated in a 0 mmol·L^−1^ Ca^2+^ solution (No Ca^2+^) containing 4 µmol·L^−1^ thapsigargin (TG), leading Ca^2+^ release from the ER (first peak). Replacement of the 0 mmol·L^−1^ Ca^2+^ solution with 1.8 mmol·L^−1^ physiological Ca^2+^ buffer (Ca^2+^) induced Ca^2+^ entry through SOC (second peak, SOCE). (**B**,**C**): Bar graphs representing the average initial slope of the ascending phase during Ca^2+^ influx (**B**) or the average maximum of Ca^2+^ influx (**C**). SOCE were recorded in 1.8 mmol·L^−1^ Ca^2+^ with the addition of DMSO (control, white bars), or YM-58483 (GBM1, GBM2 and GBM5 were treated with 1 μmol·L^−1^ and GBM3 and GBM4 with 5 μmol·L^−1^, red bars), or GSK-7975A (GBM1 and GBM2 were treated with 1 μmol·L^−1^ and GBM3, GBM4 and GBM5 with 5 μmol·L^−1^, black bars). Data represented as means ± s.e.m were obtained from recordings on at least 3 different experiments (*n* = 5 for GBM1, *n* = 3 for GBM2 and GBM3, *n* = 6 for GBM4, *n* = 4 for GBM5). The values inside the bars correspond to the numbers of recorded cells from which the data are derived. * *p* < 0.05, ** *p* < 0.01, *** *p* < 0.001, **** *p* < 0.0001. (**D**): Representative trace of the response to 1 μmol·L^−1^ of sphingosine 1-phosphate (S1P) in the absence (solid line) or presence (dashed line) of 1 μmol·L^−1^ of YM-58483 in GBM1 cells. (**E**,**F**): Quantification of the S1P (1 μmol·L^−1^) response by measuring the peak of calcium entries (maximum of response) (**E**) and with half the response time to reach the “plateau” phase (**F**) with the addition of DMSO (control, white bars, S1P), or 1 μmol·L^−1^ YM-58483 (red bars, S1P + YM-58483), or 1 μmol·L^−1^ GSK-7975A (black bars, S1P + GSK-7975A).

The ability of SOC to sustain a store-operated Ca^2+^ entry was then assessed by Ca^2+^ imaging with the ratiometric fluorescent probe Fura-2-AM that crosses the plasma membrane and binds to free cytosolic Ca^2+^. SOC activation depends on a decrease in ER Ca^2+^ concentration detected by STIM1 protein. Physiologically, Ca^2+^ leaks passively from the ER into the cytoplasm and is retrieved by the sarco-endoplasmic reticulum Ca^2+^-ATPase (SERCA) to maintain Ca^2+^ homeostasis. Irreversible inhibition of SERCA by thapsigargin (TG) results in the passive release of Ca^2+^ from the ER into the cytoplasm and thus depletes the ER of Ca^2+^. The experimental depletion of ER Ca^2+^ with TG in a Ca^2+^-free solution matches with the first peak in Figure 2A. The STIM1 protein senses the drop in Ca^2+^ in the ER, oligomerizes, and unfolds its C-terminal tail that activates SOC opening. The addition of 1.8 mmol·L^−1^ Ca^2+^ solution allows Ca^2+^ influx through open SOC, which is visualized by the second peak in Figure 2A. The speed of calcium entries (initial slope of the track, Figure 2B) and the maximum of calcium entry (maximum of normalized fluorescence ratio 340/380 nm, Figure 2C) allow the quantification of SOCE.

The results reported in Figure 2B,C show that all GSC cultures display SOCE after forced ER depletion, although with variable amplitudes. To confirm the involvement of SOC, we used YM-58483 (also called BTP2; 1 or 5 μmol·L^−1^) or GSK-7975A (1 or 5 μmol·L^−1^), both of which are SOCE inhibitors, although GSK-7975A has better specificity for blocking Ca^2+^ entry through channels of the Orai family [26]. Figure 2B,C illustrates that both YM-58483 and GSK-7975A substantially diminish Ca^2+^ influx after forced ER depletion. These data demonstrate that SOC supports Ca^2+^ entry into GSC, which we confirmed by electrophysiological recordings on GBM4 cells and GBM5 cells (Supplementary Figure S2).

Physiologically, various extracellular signals, after binding to their receptor, activate intracellular signaling pathways which in turn, stimulate SOC and ROC. Among these signals, sphingosine-1-phosphate (S1P), a molecule present in the GBM microenvironment, has been reported to induce the production of IP3 and DAG, which in turn, could trigger the activation of SOC and ROC, respectively [27,28,29,30]. Figure 2D shows that S1P leads to an increase in the intracellular Ca^2+^ concentration in GBM cells. This pattern includes two phases: a maximum of calcium entry followed by a “plateau”, resulting from Ca^2+^ influx *via* SOC and ROC. The addition of the SOCE inhibitor YM-58483 (dashed line in Figure 2D) leads to a decrease in both phases. Both the maximum response (maximum of the normalized ratio of 340/380 nm) and the half-time response (plateau phase) are affected by SOCE inhibitors (YM-58483 and GSK-7975A; Figure 2E,F), showing on the one hand the involvement of SOCE in S1P-triggered Ca^2+^ intracellular signal and on the other hand, the recruitment of SOC by physiological stimuli of the tumoral microenvironment in GSC.

### 3.2. SOCE Inhibition Reduces GSC Proliferation

**Figure 3:**
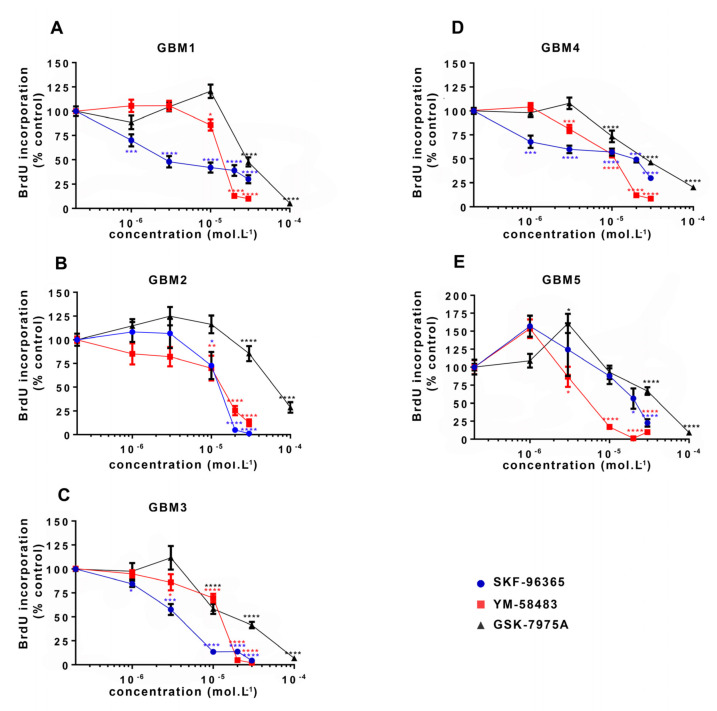
Pharmacological inhibition of SOC reduces GSC proliferation. Percentages of BrdU (bromodeoxyuridine) incorporation (expressed as percentage of control) in GSC cultures ((**A**–**E**): GBM1-GBM5) maintained for 24 h with increasing concentrations of SKF-96365 (blue), YM-58483 (red) or GSK-7975A (black). Data represent means ± s.e.m of four independent experiments, with each condition assessed in quadruplicate per experiment. * *p* < 0.05, ** *p* < 0.01, *** *p* < 0.001, **** *p* < 0.0001.

Initially, the involvement of SOCE in GSC proliferation was directly evaluated by subjecting cells to BrdU incorporation after 24 h treatment with SOC inhibitors. For this ELISA BrdU incorporation assay, the two SOC inhibitors YM-58483 and GSK-7975A were used as well as SKF-96365, previously used to inhibit SOCE in GSC [31]. Figure 3 shows that all SOC inhibitors dose-dependently reduced GSC proliferation in all cultures, with a maximal effect observed at the concentration of 30 μmol·L^−1^ for SKF-96365 and YM-58483, and 100 μmol·L^−1^ for GSK-7975A.

In order to determine a possible effect of SOC inhibitors on cell death, the proportion of dead cells was determined using a viability assay based on the use of Calcein-AM, to detect live cells, and ethidium homodimer-1 (EthD-1), to label dead cells. This assay performed on GBM1, GBM4 and GBM5 cells showed that the percentage of dead cells in GSC cultures was only slightly affected by 24 h-treatment with SKF-96365, YM-58483 and GSK-7975A, even with the highest concentrations of the drugs, which increased cell death by approximately 10% as compared to control (Supplementary Figure S3). These results, in combination with the data from BrdU assay, support the fact that SOCE inhibition results in decreased proliferation of GSC.

### 3.3. SOCE Inhibition Reduces the Stem Cell Population in GSC Cultures

**Figure 4:**
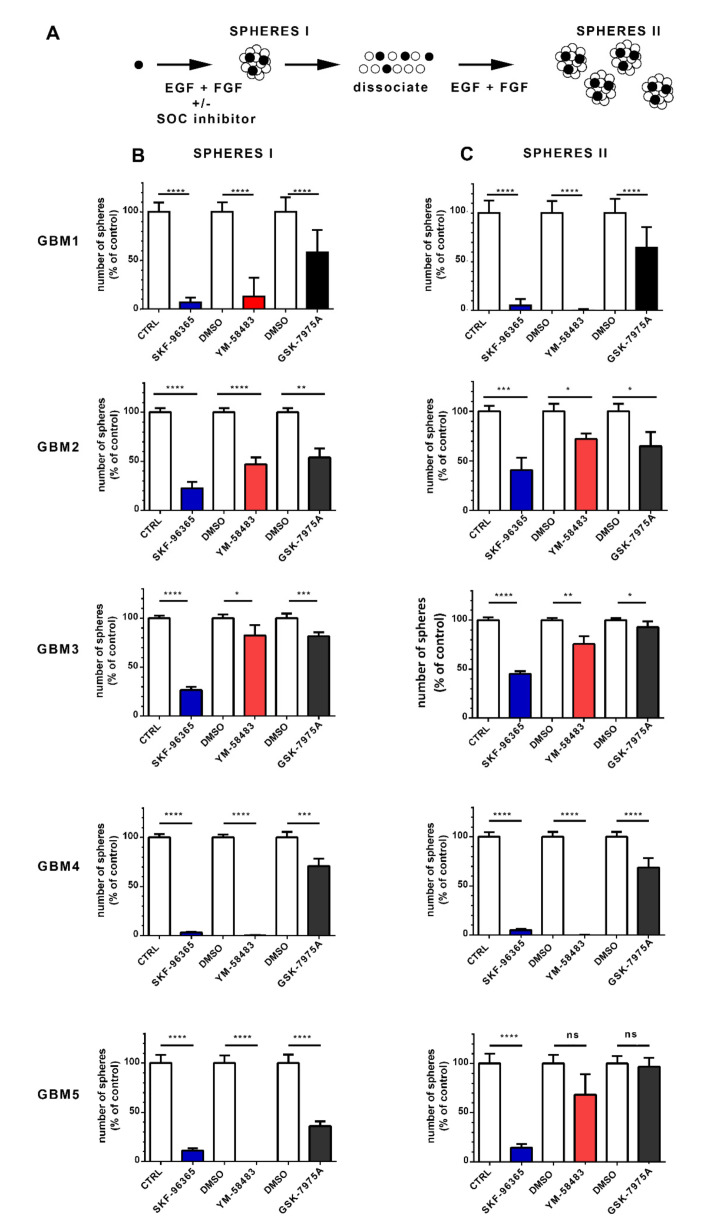
Pharmacological inhibition of SOC impairs the ability of GSC to form sphere and self-renew. (**A**): Schematic of the assay used to obtain the data in B and C. Stem cells represented by black circles proliferate and form spheres (SPHERES I) that contain both stem cells (black) and more differentiated cells (white circles). Following dissociation of sphere I cultures, cells with stem cell capacity will form new spheres (SPHERES II) when replated with growth factors, allowing the assessment of the stem cell population that was present in the sphere I cultures. This analysis was performed on GBM1, GBM2, GBM3, GBM4 and GBM5 cells. (**B**): Number of primary spheres ((**B**), SPHERES I) obtained following 1 week of culture with either 3 μmol·L^−1^ SKF-96365 (blue bars), 10 μmol·L^−1^ YM-58483 (red bars) or 20 μmol·L^−1^ GSK-7975A (black bars), compared with their controls (white bars, water for SKF-96365, and DMSO for YM-58483 and GSK-7975A). Data are expressed as percentage of control and represent means ± s.e.m of three independent experiments, with each condition assessed in triplicate per experiment: * *p* < 0.05, ** *p* < 0.01, *** *p* < 0.001, **** *p* < 0.0001. (**C**): Sphere I cultures obtained under different conditions in B were collected, dissociated as single cells and plated at a similar concentration in culture medium containing only growth factors (without SOC inhibitor). This resulted in new spheres (secondary spheres, SPHERES II), the number of which is shown in (**C**). Data are expressed as percentage of control and represent means ± s.e.m of three independent experiments, with each condition evaluated in triplicate per experiment. ns: not significant, * *p* < 0.05, ** *p* < 0.01, *** *p* < 0.001, **** *p* < 0.0001.

GSC are characterized by their ability to form spheres in vitro when maintained in a defined medium containing the growth factors EGF and FGF. To determine whether SOC are involved in the ability of GSC to form spheres, we exposed GSC for 7 days to SKF-96365, YM-58483 or GSK-7975A. At the end of this period, the number of primary spheres, reflecting the GSC stem cell activity, was counted (Figure 4A SPHERES I). While a single GSC may be the origin of a sphere, each sphere is composed of both GSC and more differentiated cells. To obtain more insights into the possible effects of drugs on the GSC population in cultures after treatments, the cells were collected, dissociated, and then reseeded at the same number in fresh culture medium without SOC inhibitors to determine sphere formation after 7 days (Figure 4A SPHERES II). This number reflects the number of GSC present in the culture after treatment with SOC inhibitors and provides insight into the impact of treatment GSC self-renewal.

For this assay, SKF-96365, YM-58483 and GSK-7975A were used at 3 μmol·L^−1^, 10 μmol·L^−1^ and 20 μmol·L^−1^, respectively, which still allowed substantial cell proliferation (at least 50% of cell proliferation compared with control) and thus permitted the development of spheres. As shown in Figure 4B, all SOC inhibitors decreased the formation of primary spheres and thus substantially restrained the stem-like cell activity of GSC. Specifically, SKF-96365 and YM-58483 displayed dramatic effects in nearly all GBM cultures, whereas GSK-7975A, which preferentially targets the Orai family [26], exerted less pronounced effects (Figure 4B SPHERES I), implying that TRPC1, in conjunction with Orai1, may contribute to the effects of SOC in GSC.

The GSC cultures obtained after the different treatments were then collected, dissociated and plated as single cells at identical concentrations but without inhibitors to determine the proportion of GSC after the different treatments. Figure 4C (SPHERES II) shows that the cells obtained from primary sphere cultures in the presence of SOC inhibitors formed fewer secondary spheres than the cells maintained under control conditions, indicating that the stem cell population was decreased in cultures that had been treated with SOC inhibitors. These data suggest that SOC inhibition hampers the self-renewal ability of GSC.

**Figure 5:**
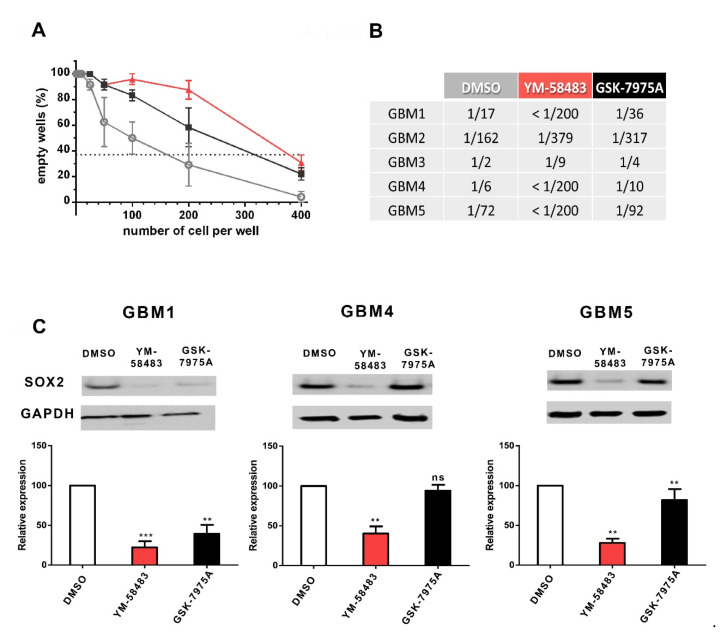
Pharmacological inhibition of SOC reduces the stem cell population and expression of SOX2 stem cell marker. (**A**,**B**): GSC frequency evaluated by a limit dilution assay. Cells were grown in their medium for 7 days with 10 μmol·L^−1^ YM-58483 (red), 20 μmol·L^−1^ GSK-7975A (black), or DMSO (grey) at the concentrations of 400 to 1 cell per well. After one week, wells without spheres were counted for each cell density. Graph A illustrates the data obtained in GBM2. According to Poisson’s law, the number of cells per well where 37% of the wells are empty corresponds to the frequency of GSC. Table B provides the data obtained in all GBM cultures. These data indicate the frequency of cells capable of forming a sphere. For example, the frequency of cells capable of forming a sphere in the control condition (DMSO) for GBM1 is 1/17, meaning that among 17 cells, only one will form a sphere. (**C**): SOX2 expression in GBM1, GBM4 and GBM5 GSC cultures maintained during 7 days with 10 μmol·L^−1^ YM-58483, 20 μmol·L^−1^ GSK-7975A, or DMSO. An example blot is shown in the upper part of the figure, and the quantifications obtained on 5 to 7 experiments are indicated. White bars correspond to control conditions (DMSO), red bars to YM-58483-treated cultures and black bars to GSK-7975A-treated cells. ns: not significant, ** *p* < 0.01, *** *p* < 0.001.

The effect of SOCE inhibitors on GSC was confirmed using a limit dilution assay (Figure 5A) that quantifies GSC in cultures by their capacity to form spheres. An example of this assay is shown in A, and the quantifications are given in the table in Figure 5B. The results of this assay for GBM1 cells, for example, show that in the control condition, 1 out of 17 cells is endowed with the capacity to form a sphere. The addition of YM-58483 or GSK-7975A substantially decreased the frequency of cells exhibiting this GSC property to, respectively, less than 1/200 or 1/36 cells, meaning that only 1 cell out of more than 200 cells in YM-58483 condition and only 1 out of 36 cells in GSK-7975A condition will form a sphere, whereas in control condition 1 out of 17 cells forms a sphere. Thus, in the presence of either YM-58483 or GSK-7975A, the stem cell capacity is substantially reduced. The table in Figure 5B illustrates that, although the frequency of cells forming spheres was different in cultures under control conditions (DMSO), YM-58483 and GSK-7975A reduced the frequency of GSC in all GBM cultures.

To further confirm the effects of SOC inhibitors on the stem cell population, the expression of SOX2 was assessed. SOX2, which is a transcription factor necessary for the maintenance of glioblastoma stem cell tumorigenic activity, is commonly used to detect the stem cell population in glioblastoma [32,33,34]. Figure 5C illustrates that SOX2 expression was significantly decreased upon treatment with YM-58483 in all GBM cells tested and with GSK-7975A in 2 of 3 GBM cells tested, confirming a role of SOC in the maintenance or expansion of GSC.

## 4. Discussion

Using five different patient-derived tumor-cultures, we demonstrate that GSC express SOC proteins that functionally sustain Ca^2+^ entry and participate in S1P-induced intracellular Ca^2+^ signaling. Pharmacological inhibition of SOC diminishes proliferation and substantially reduces the self-renewal capacities of GSC. Our study thus identifies a pivotal role of SOC in the maintenance or expansion of the stem cell pool in GBM. A previous transcriptomic analysis suggested a major role for Ca^2+^ signaling in these cells [6]. Our study clarifies that Ca^2+^ entry through SOC represents a major mechanism involved in the stemness maintenance of GSC, considered to be responsible for the aggressiveness of GBM. As we identified SOC as major actors in GSC, we analyzed large-scale, publicly available databases (http://gliovis.bioinfo.cnio.es/ (accessed on 22 February 2021)) to explore whether TRPC1, Orai1 or STIM1 correlate to GBM outcome [35]. We observed that deregulation of SOC actors is associated with significantly reduced overall patient survival (Supplementary Figure S4). Specifically, elevated levels of Orai1 or STIM1 are associated with decreased patient survival. As the presence of GSC in brain tumors worsens the outcome of GBM patients, the present demonstration of a role for SOC in GSC identifies a possible molecular target for reducing the aggressiveness of GBM.

Since the identification of GSC in GBM cell cultures, much progress has been made in characterizing the extracellular signals that influence their proliferation and stemness, but the mechanisms involved in the integration of the different signals remain unknown. Ca^2+^ signaling pathways have been identified as a prominent pathway in GSC based on the analysis of the transcriptomic signature of these immature GBM cells compared to more differentiated GBM cells [6]. Among Ca^2+^ channels, SOC have the capacity to mobilize a long-lasting Ca^2+^ influx in response to multiple cell surface receptor stimulations. They are recruited by various extracellular signals such as HGF or S1P, both of which are major drivers of glioma progression and promote stemness in glioblastoma cells [27,28,36,37,38,39]. Our study shows that GSC from patient-derived surgical tumor specimen express Orai1 and TRPC1 as well as STIM1, as do the adult neural stem cells from which GSC originate, at least in part [16,17,18,40,41,42,43,44]. Using Ca^2+^-imaging approaches, we demonstrate that SOC are functional within GSC as they are able to generate Ca^2+^ entry after forced depletion of the ER store. To determine whether SOC identified in GSC can be recruited by physiological signals, we exposed the cells to S1P, a signal known to recruit PLC, which leads to the formation of IP3 that, in turn, triggers the release of Ca^2+^ from the ER and the activation of SOC. Physiologically, S1P can activate both ROC and SOC. While we cannot rule out a possible role of ROC in the response to S1P, our data showing that two different SOC inhibitors reduce S1P-induced Ca^2+^ entry support the idea that GSC possess SOC that can be activated by signals from the tumor microenvironment [28]. Thus SOC-mediated Ca^2+^ influx may represent, for GSC, key players for transduction and possibly integration of information from the tumor microenvironment.

To better understand their pathophysiological roles in GSC, SOC were inhibited by pharmacological drugs, which resulted in impaired proliferation in cultures. This effect has also been described in conventional GBM cell lines [13,14,45] and in primary cultures of GSC derived from GBM of patients that were maintained with growth factors and without fetal bovine serum [31]. In the latter study, the inhibition of Ca^2+^ entry with SKF-96365 led to a shift of GSC toward a quiescent state [31]. Interestingly, primary cultures from patients maintained in a culture medium containing fetal bovine serum, a culture condition known to induce stem cell differentiation, were only marginally affected by SOC inhibition [14]. Our results, along with these data, suggest that cells in a stem cell state may be sensitive to SOC-mediated Ca^2+^ influx.

Our study provides evidence that inhibition of Ca^2+^ influx by SOC inhibitors reduces the stem cell population in GSC cultures, a process that is accompanied by a decrease in the SOX2 stem cell marker expression. This decrease in stem cell population was observed with concentrations of SKF-96365 and YM-58483 that have been used to block the physiological effects of SOC [31,46,47,48,49]. As SKF-96365 and YM-58483 may also target other receptor channels (ROC), GSK-7975A, a more specific inhibitor of Orai-dependent SOCE was used [50,51,52]. The fact that GSK-7975A had also significant effects on GSC supports the idea that SOC play a key role in maintaining the GSC pool in GBM as they do in oral/oropharyngeal squamous cell carcinoma cancer stem cells and in liver cancer stem cells [53,54]. How SOC maintain the cancer stem cell population remains unknown. One possibility is that SOC affect the mode of GSC division. GSC can divide in two modes. The symmetric proliferative expansion mode will produce two GSC and thus increase the GSC pool in the tumor, whereas asymmetric cell division will generate one GSC and one non-GSC cell and thereby maintain the GSC pool. We have previously observed that SOC inhibitors decrease symmetric cell division of subventricular neural stem cells, which are possible cells of origin of GBM [17]. If SOC exert the same effect in GSC, SOC inhibition will decrease the symmetrical cell division in GSC, which should lead to a reduction in self-renewal and a decrease in the stem cell population, as has been found in GSC treated with BMP4, for example [55].

Interestingly, a recent study identified Ca^2+^ as a guardian of pluripotency in embryonic stem cells [56]. Our present demonstration of a role for SOC in GSC consolidates the idea that Ca^2+^ may be essential for stemness, and further suggests that SOC represent an essential Ca^2+^ handling mechanism involved in stemness maintenance, in both physiological and cancer stem cells [17,53,54,57]. SOC-mediated Ca^2+^ entry has been identified to activate Ca^2+/^calmodulin-dependent protein kinase II (CaMKII) as well as well as NFAT [58,59,60,61]. Interestingly, the inhibition of CaMKII or of NFAT reduced stemness in GBM cell cultures [62,63,64], indicating that Ca^2+^ signaling plays a key role in maintaining or expanding the subpopulation of tumor-initiating cells responsible for GBM relapse. Several reports have identified Ca^2+^ channels as essential in different cancer stem cells, although the specific roles of SOC remain to be clarified [65,66]. This central role of Ca^2+^ is not restricted to stem cells but also relates to overall tumor progression, which has led to suggestions that Ca^2+^ dyshomeostasis is a hallmark of cancer [67,68]. Thus, it would be of interest to design future studies to determine the extent to which Ca^2+^ dyshomeostasis [10,14] affects GSC activities and to fully explore the Ca^2+^ toolkit involved in GBM initiation, progression and recurrence.

## 5. Conclusions

Our study based on multiple approaches demonstrates that GSC in primary cultures derived from GBM patients express functional SOC whose pharmacological inhibition impairs proliferation as well as self-renewal ability. As GSC are responsible for GBM resistance to treatment, and given that experimental phase II cancer therapies with molecules that target a wide range of Ca^2+^ channels are tolerated by—and ameliorate survival of—patients with GBM [69], our study will help to nurture the framework for identifying new therapies against this deadly pathology.

## Data Availability

Data are reported in figures and in supplementary data online.

## Supplementary Materials

The following are available online at https://www.mdpi.com/article/10.3390/cancers13143428/s1: Table S1: Primary antibodies used in the study, Figure S1: Expression of CD44 or CD133 in glioblastoma cancer stem cells, Figure S2: I/V relationships of currents from GBM4 and GBM5 cells, Figure S3: Cell death in glioblastoma stem cell cultures, Figure S4: Survival analysis using the CGGA, TCGA and Gravendeel database (from the GlioVis database, http://gliovis.bioinfo.cnio.es/, accessed on 1 July 2021), whole blots of Figure 1 and Figure 5.

## Abbreviations

DAG: diacylglycerol; ER: Endoplasmic Reticulum; GBM: glioblastoma; GSC: Glioblastoma Stem Cells; IP3: inositol triphosphate; PIP2: phosphatidylinositol-4,5-bisphosphate; PKC: protein kinase C; ROC: Receptor-operated channels; ROCE: Receptor-operated Ca^2+^ entries; S1P: Sphingosine-1-Phosphate; SERCA: sarco-endoplasmic reticulum Ca^2+^-ATPase; SOC: Store-operated Channels; SOCE: Store-operated Ca^2+^ entries; STIM: Stromal Interaction Molecule; TG: thapsigargin; TRPC: Transient Receptor Potential Canonical.
