# Supplementary material for: Store-Operated Calcium Channels Control Proliferation and Self-Renewal of Cancer Stem Cells from Glioblastoma
Source: Cancers (Basel). 2021 Jul 8;13(14):3428. doi: 10.3390/cancers13143428 (PMC8307764; doi:10.3390/cancers13143428)
Supplement: Supplementary file 1 [file cancers-13-03428-s001.zip › cancers-1264888-supplementary.pdf]

Supplementary data of the manuscript entitled

Store-operated calcium channels control proliferation and self-renewal of cancer stem cells from glioblastoma

By

Elodie TERRIÉ[1], Nadine DELIOT[1], Yassine BENZIDANE[1], Thomas HARNOIS[1], Laëtitia COUSIN[1], Patrick BOIS[2], Lisa OLIVER[3], Patricia ARNAULT[1], François VALLETTE[3,4], Bruno CONSTANTIN[1,4], Valérie CORONAS[1,4]

This file contains a Supplementary Table (Supplementary Table 1) and 4 Supplementary Figures (Supplementary Figures 1-4)

Supplementary Table 1: Primary antibodies used in the study.

Antigen	Source	Origin	Reference	Dilution	
				WB	IF
TRPC1	Santa Cruz Biotechnology	mouse monoclonal	sc-133076	0.2 $\mu g.ml^{-1}$	2 $\mu g.ml^{-1}$
Orai1	Santa Cruz Biotechnology	rabbit polyclonal	sc-68895	0.2 $\mu g.ml^{-1}$	0.4 $\mu g.ml^{-1}$
STIM1	BD Biosciences	mouse monoclonal	610954	0.25 $\mu g.ml^{-1}$	2 $\mu g.ml^{-1}$
SOX2	Santa Cruz Biotechnology	mouse monoclonal	sc-365964	0.2 $\mu g.ml^{-1}$	2 $\mu g.ml^{-1}$
SOX2	Santa Cruz Biotechnology	goat polyclonal	sc-17320		1 $\mu g.ml^{-1}$
CD133	Merck Millipore	mouse monoclonal	MAB4399-I		10 $\mu g.ml^{-1}$
CD44	BD Biosciences	mouse monoclonal conjugated with FITC	347943		1.25 $\mu g.ml^{-1}$
GAPDH	Santa Cruz Biotechnology	mouse monoclonal	sc-32233	0.1 $\mu g.ml^{-1}$	

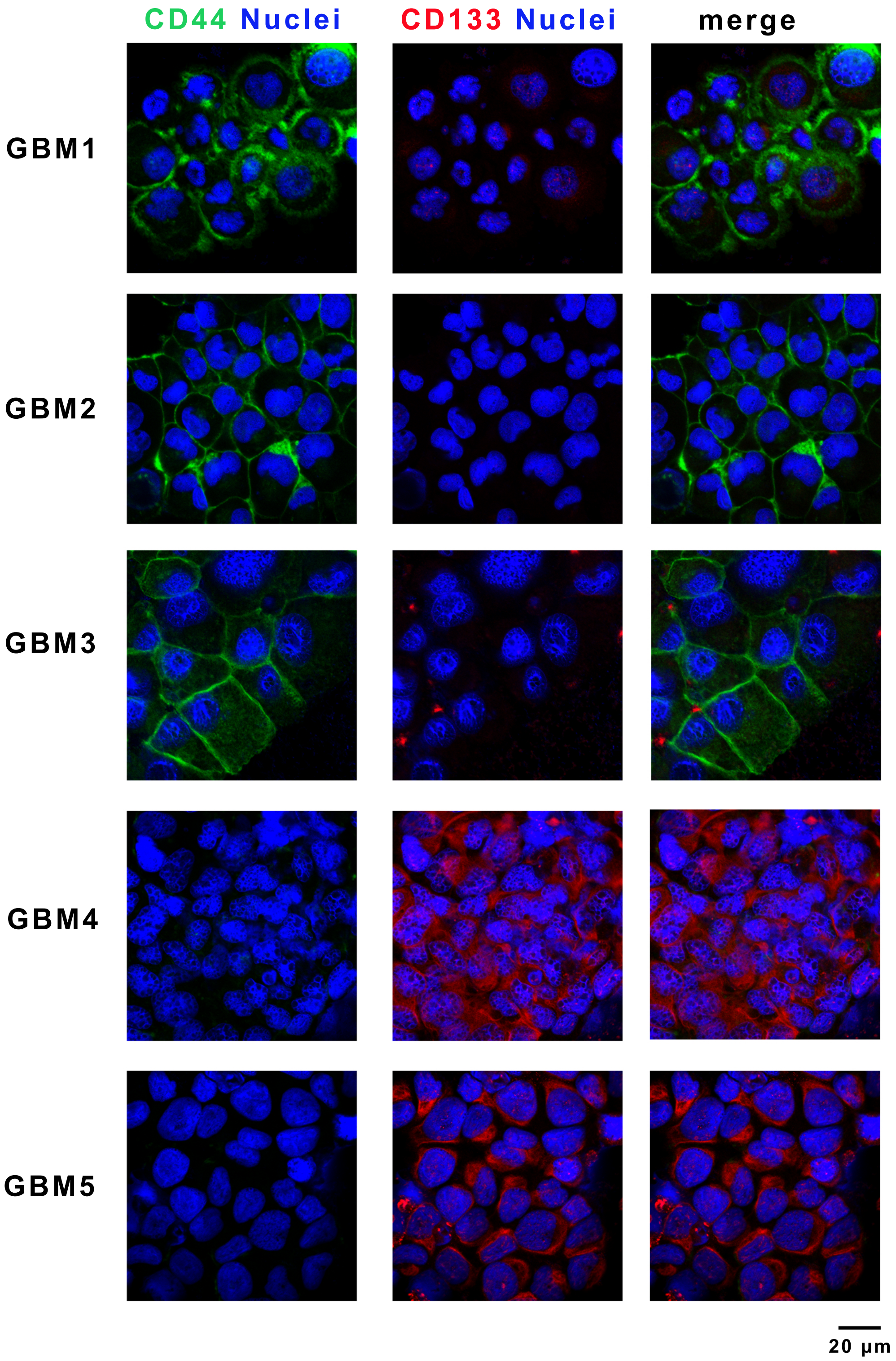

Supplementary Figure 1: Expression of CD44 (green) or CD133 (red) in glioblastoma cancer stem cells used in the study. Nuclei are labelled in blue with TOPRO-3.

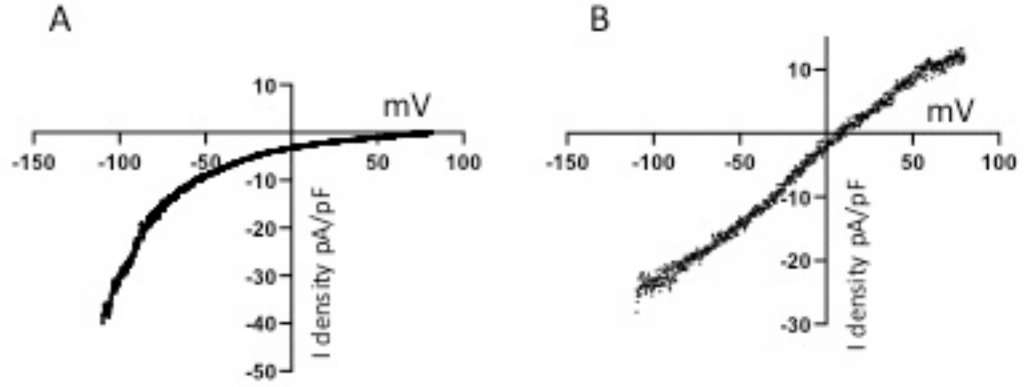

Supplementary Figure 2: I/V relationships of currents from GBM4 and GBM5 cells

The current-voltage I/V curve was obtained by the difference between the ramp currents recorded in the Ca^{2+} free solution and the divalent-free (DVF) solution. Note the important net inward current with large inward rectification caused by the conductance of the monovalent cation (Na+). The I/V properties of GMB4 cells are characteristic of the CRAC current. **B.** In a different way, I/V curve obtained on GBM5 cells does not show any rectification. This absence of rectification is distinguishing feature of the TRPC channels.

The I_{CRAC} current and “TRPC like” currents were recorded on 5 other cells. The estimated density for GBM4 and GBM5 cells, are near 40 pA/pF and 20 pA/pF, respectively.

Patch-clamp method : To obtain the above results, SOCE currents were measured by patch-clamp recordings in whole-cell configuration by using a protocol adapted from Alansari et al 2014 (Measuring endogenous I_{CRAC} and ORAI currents with the patch-clamp technique. Cold Spring Harb Protoc 2014 (6):630-7. doi: 10.1101/pdb.prot073254). The signals were recorded with an Axopatch 200B amplifier with a CV 203BU headstage (Molecular Devices, CA). Voltage command pulses were generated by a personal computer equipped with an analog-digital converter (Digidata 1322A; Molecular Devices) using pCLAMP software v10.0

(Molecular Devices). Patch pipettes were pulled from borosilicate glass capillary tubes (GC 150T-10; Clark Electromedical Instruments, Harvard Appartus Edenbridge, UK) using a vertical micropipette puller (Narishige, Tokyo, Japan) and had resistances ranging from 2 to 3 MΩ when filled with internal pipette solution following composition (in mmol.l^{-1}):105 Cs-methanesulfonate, 20 Cs-1,2-bis-(2-aminophenoxy)ethane-N,N,N′,N′-tetraacetic acid (Cs-BAPTA), 8 MgCl2, and 10 HEPES (pH adjusted to 7.2 with CsOH). BAPTA was used to generate a Ca^{2+} store depletion. To amplify the SOCE current (which is mainly, in this protocol, carried by sodium), the bath solution was switched from a Ca^{2+} free solution containing (in mmol.l^{-1}) 145 Cs-methanesulfonate, 20 Cs-Cl, 1,2 Mg SO4, and 10 HEPES (pH adjusted to 7.2 with NaOH) to a divalent-free (DVF) solution containing (in mM) 135 Na-methanesulfonate, 10 HEDTA, 1 EDTA, and 10 HEPES (pH 7.4, adjusted with NaOH). The SOCE current was recorded after application of voltage ramps from -110 mV to +80 mV within 1 s every 5 s. Data were analyzed using Clampfit software v10.4 (Molecular Devices).

A

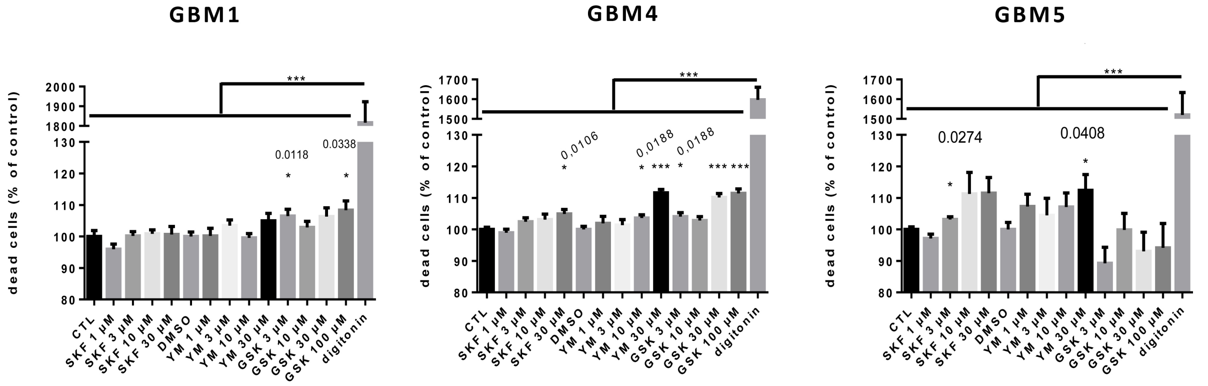

B

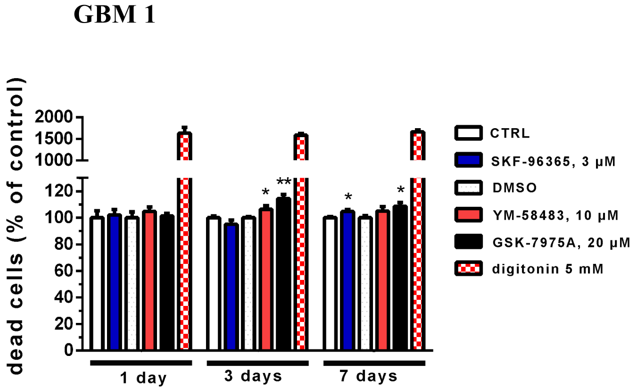

Supplementary Figure 3: Cell death in glioblastoma stem cell cultures following exposure to SOC inhibitors. Percentage of dead (Ethidium bromide positive) cells compared to control (in the absence of the drugs) on cultures maintained for 24h (in A) with increasing concentrations of SKF-96365 (SKF), YM-58483 (YM) or GSK-7975A (GSK) or with 3 µmol.l^{-1} of SKF-96365 (in blue), 10 µmol.l^{-1} YM-58483 (in red) or 20 µmol.l^{-1} GSK-7975A (in black) during 1, 3 or 7 days (in B). Digitonin (5 mmol.l^{-1}) was added to some cells during the last hour of culture as a positive control of cell death. The data, expressed as percentage of control, represent means ± s.e.m of three independent experiments, with each condition assessed in triplicate per experiment. * P<0.05, ** P< 0,01, *** P<0.0001.

Method used for cell death assay. To obtain the above results, cell death was evaluated using the staining markers Calcein-AM and Ethidium homodimer-1 (EthD-1) for live and dead cells respectively (Sigma Aldrich (Saint-Louis, USA). GBM spheres were dissociated, seeded in a

96 well-plate at 10000, 5000 or 2000 cells per well and treated with SOC inhibitors for 24 hours, 72 hours or 7 days. At the end of the treatment period, the cells were stained with 3.10^{-6} $mmol.l^{-1}$ Calcein-AM and 1.10^{-5} $mmol.l^{-1}$EthD-1. Fluorescences were read separately using FleXstation: for Calcein-AM, the excitation was at 485 nm and the emission at 530 nm and for EthD-1, the excitation was at 530 nm and the emission at 645 nm.

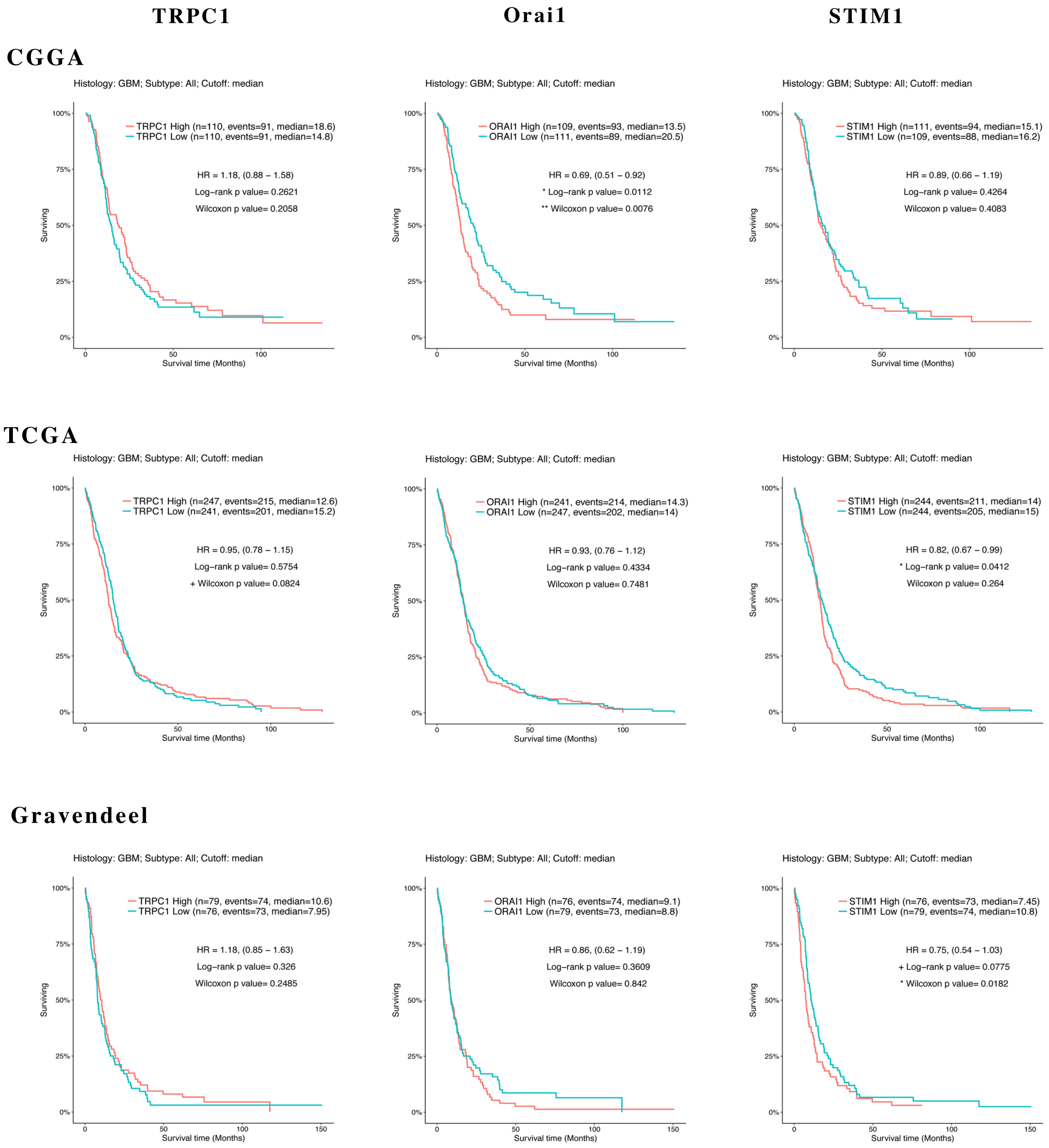

Supplementary Figure 4: Survival analysis using the CGGA, TCGA and Gravendeel database showing that high expression (trace in red) of Orai1 (for CGA database) or STIM1 (for TGCGA and Gravendeel database) is associated with significant decreased survival of patients with GBM. Data are from http://gliovis.bioinfo.cnio.es/
